# Safety of the CSF1R Inhibitor EI‐1071 in Human and Its Pharmacological Effects to Reduce Neuroinflammation and Improve Memory Function in a Mouse Model of Alzheimer’s Disease

**DOI:** 10.1002/alz.091161

**Published:** 2025-01-09

**Authors:** Wahyu Dewi Tamayanti, Jun Ru Lin, Agnes Dwi Ariyanti, Yenni Chang, Haw Yuan Cheng, Chia Wei Huang, Wu Cheng Ru, Chuan Yao Wang, Tsung Han Hsieh, Uni D Lin, Chi Yun Pai, Jin‐Wu Tsai, Hung‐Kai Chen

**Affiliations:** ^1^ Institute of Brain Science, Schools of Medicine, National Yang‐Ming Chiao Tung University, Taipei, Taiwan, Taipei City Taiwan; ^2^ Elixiron Immunotherapeutics, Taipei City Taiwan; ^3^ Institute of Brain Science, Schools of Medicine, National Yang‐Ming Chiao Tung University, Taipei, Taiwan, Taipei Taiwan; ^4^ Elixiron Immunotherapeutics, Taipei Taiwan

## Abstract

**Background:**

Microglial activation is one of the neuropathological hallmarks of Alzheimer’s disease (AD). Evidence suggest that chronic activation of microglia cause neuroinflammation and neuronal injuries, contributing to cognitive impairment. Therefore, modulation of microglial pathway like CSF‐1R represents an attractive therapeutic strategy. EI‐1071 (enrupatinib) is a brain‐penetrant CSF‐1R selective inhibitor that is in clinical development for AD treatment. Here we report the pharmacological effects of EI‐1071 on microglial modulation and neuronal functions in the 5xFAD model and the safety/tolerability demonstrated in a first‐in‐human trial.

**Method:**

In animal studies, neurons in brains of 5xFAD mice were marked with EGFP via *in utero* electroporation. Mice with/without EI‐1071 treatment were assessed by behavioral studies including Novel Object Recognition (NOR) and Y‐maze followed by microscopic examination of Aβ plaques and GFP‐labelled neurons in brain slices. Changes in microglia and inflammatory biomarkers were analysed by immunofluorescent microscopy, RNA sequencing and/or quantitative PCR. A first‐in‐human phase 1 trial (NCT04238364) was conducted in healthy volunteers in compliance with the protocol approved by the regulatory authorities.

**Result:**

Pharmacological inhibition of CSF‐1R by EI‐1071 induced significant changes in microglial homeostasis, neuroinflammation, and neuronal injuries. Those activated microglia surrounding amyloid plaques were greatly reduced accompanying with a declining tendency in amyloid plaques and neuronal death. Treatment of EI‐1071 did not eliminate all microglia. Homeostatic microglia were relatively well preserved as compared to the plaque‐associated microglia. Gene expression analysis revealed declines in microglial and proinflammatory genes and AD‐associated biomarkers including *Iba1*, *Csf1r*, *Trem2* and *Tyrobp*. In addition, mice treated with EI‐1071 showed enhanced cognitive functions in NOR and Y maze tests.

The phase 1 study enrolled 58 healthy volunteers into the single ascending dose and multiple ascending dose phases to assess the safety and tolerability of EI‐1071. All treatment emergent adverse events in the multiple dosing phase were grade 1, and were unrelated to EI‐1071 dosing.

**Conclusion:**

1. EI‐1071 is effective in reducing activated microglia, neuroinflammation and neuronal injuries, resulting in a significant improvement of memory function in the 5xFAD model.

2. EI‐1071 is well‐tolerated in a phase 1 clinical trial. The safety profile supports further investigation in patients with AD.

